# Cardiac Denervation and Reinnervation After Heart Transplantation: Assessment and Clinical Impact

**DOI:** 10.3390/medicina62101987

**Published:** 2026-10-14

**Authors:** Antonella Galeone, Gina Mazzeo, Michele Moretti, Enrico Munari, Alessia Gambaro, Teresa Maria Capovilla, Matteo Brunelli, Giovanni Battista Luciani

**Affiliations:** 1 Department of Surgery, Dentistry, Pediatrics and Gynecology, Division of Cardiac Surgery, University of Verona, 372126 Verona, Italy; 2 Division of Cardiology, Santa Chiara Hospital, ASUIT, 38123 Trento, Italy; 3 Department of Public Health and Diagnsotics, Division of Pathology, University of Verona, 37126 Verona, Italy; 4 Division of Cardiology, Azienda Ospedaliera Universitaria Integrate of Verona, 37126 Verona, Italy; 5 Division of Cardiology, Santa Maria Del Carmine Hospital, ASUIT, 38068 Rovereto, Italy

**Keywords:** heart transplantation, cardiac denervation, cardiac reinnervation, heart rate variability, cardiac allograft vasculopathy, myocardial scintigraphy

## Abstract

The heart is innervated by an intricate network involving sympathetic, parasympathetic, and sensory afferent pathways, which together regulate heart rate, contractility, and vascular tone in response to physiological demands. This innervation is anatomically organized along the neurocardiac axis, from the intrinsic cardiac ganglia to central autonomic centers, and molecularly shaped during development by neurotrophic and repulsive signaling cues. Orthotopic heart transplantation inevitably severs all afferent and efferent neural connections between the donor heart and the recipient, producing a state of complete cardiac denervation that manifests as resting tachycardia, blunted heart rate variability, and abolished reflex chronotropic responses. Over time, a variable degree of sympathetic and, less consistently, parasympathetic reinnervation may occur in a subset of recipients, predominantly in the anterior wall of the left ventricle, typically beginning after the first post-transplant year and progressing gradually, with a characteristic base-to-apex and epicardium-to-endocardium pattern that often remains incomplete even at long-term follow-up. Reinnervation is currently assessed through complementary non-invasive approaches: heart rate variability analysis provides an indirect functional readout of autonomic modulation, while nuclear imaging techniques (including ^123^I-metaiodobenzylguanidine scintigraphy and ^11^C-metahydroxyephedrine positron emission tomography) allow direct visualization and quantification of regional sympathetic nerve density and integrity. The extent and pattern of reinnervation appear clinically relevant, as denervation and its recovery have been associated with exercise capacity, arrhythmic risk, silent myocardial ischemia, and long-term prognosis after transplantation, although findings across studies remain heterogeneous. This narrative review summarizes current knowledge of cardiac autonomic anatomy and neurotransmission, the molecular determinants of innervation patterning, the physiological consequences of heart transplant-related denervation, the evidence on post-transplant reinnervation and its non-invasive assessment, and the impact of reinnervation on clinical outcomes.

## 1. Introduction

The heart and the brain are linked through a complex bidirectional communication network. The heart ensures continuous cerebral perfusion, while the brain modulates heart function via neural mechanisms. Cardiac activity is modulated by the sympathetic and parasympathetic branches of the peripheral autonomic nervous system, in addition to afferent sensory inputs [1]. Sympathetic neurons predominantly release norepinephrine (NE) as their principal neurotransmitter, whereas acetylcholine (ACh) is the main mediator released by both preganglionic and postganglionic fibers of the vagus nerve, thereby driving parasympathetic activation. A number of radiotracers employed in cardiac imaging are structural analogs of neurotransmitters, particularly NE, enabling the non-invasive evaluation of cardiac sympathetic innervation through nuclear imaging modalities [2]. The sympathetic and parasympathetic systems exert opposing influences on cardiac function, finely tuning heart rate (HR) and myocardial contractility. Sympathetic stimulation enhances HR (positive chronotropic effect) and increases contractile force (positive inotropic effect), while parasympathetic activation reduces HR (negative chronotropy) and counteracts sympathetic-mediated contractile responses. Under normal physiological conditions, a dynamic balance between these two systems ensures appropriate cardiovascular adaptation to internal and external demands. Heart transplantation (HT) results in complete denervation of the donor heart with loss of sympathetic and parasympathetic regulation. The re-innervation process usually occurs after the first year post-transplant and involves myocardial muscle, sinoatrial (SA) node, and coronary vessels [3]. Multiple diagnostic techniques may be used to show the reinnervation of the transplanted heart including assessment of the heart rate variability (HRV), cardiopulmonary exercise testing (CPET), immunohistochemistry and myocardial scintigraphy (Table 1). The re-innervation process is progressive but incomplete and may affect survival and enhance the development of cardiac allograft vasculopathy (CAV) in heart recipients. The aim of this narrative review is to discuss the current available techniques used to evaluate cardiac innervation after HT and the effect of cardiac denervation on heart recipients’ morbidity and mortality.

## 2. Anatomy of the Autonomic Innervation of the Heart

**Figure 1:**
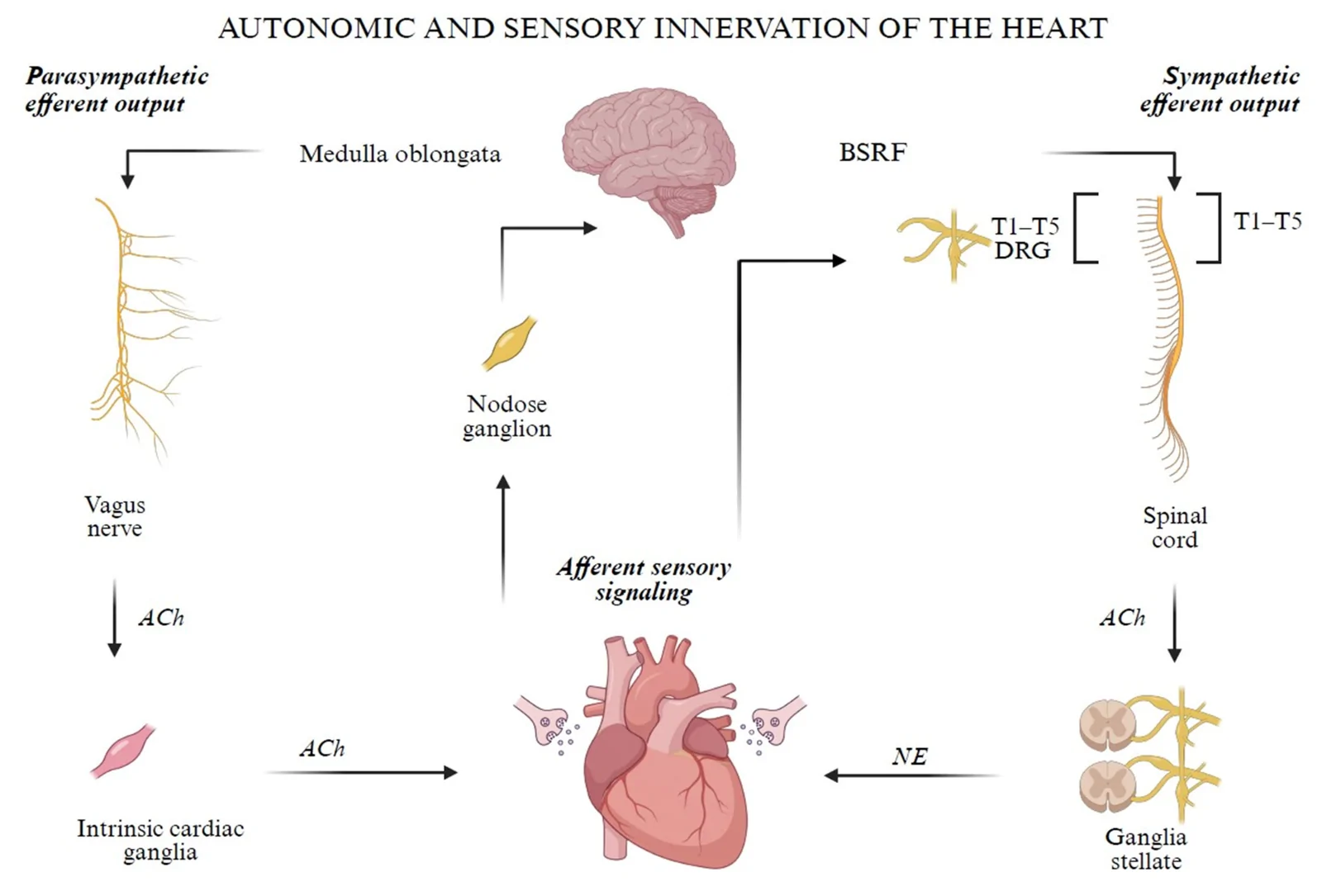
Efferent and afferent cardiac innervation. ACh: acetylcholine; BSRF: brainstem reticular formation; DRG: dorsal root ganglia; NE: norepinephrine.

Growing evidence over the past decades demonstrated that the autonomic regulation of the heart is highly complex. The cardiac autonomic nervous system comprises a network of interacting feedback circuits involving intrinsic cardiac ganglia, extracardiac intrathoracic ganglia, the spinal cord, the brainstem, and higher central nervous system centers (Figure 1). Through this hierarchical and integrated network, neural control modulates both cardiac excitability and contractile function. Neurons contributing to cardiac regulation are distributed along the entire neurocardiac axis, extending from cortical regions such as the insular cortex to the heart [4]. At the organ level, this regulation is mediated by the intrinsic cardiac nervous system, a distributed network of ganglia interconnected by local neural pathways [5,6].

### 2.1. Parasympathetic Innervation of the Heart

The medulla oblongata regulates sympathetic and parasympathetic outflow to the heart and blood vessels. The parasympathetic control of the heart is primarily mediated by the vagus nerve, which originates in the brainstem at the medulla oblongata. The vagus nerve has three nuclei associated with cardiovascular control: the dorsal motor nucleus, the nucleus ambiguus, and the solitary nucleus, which receives sensory input about the state of the cardiovascular system as an integrating hub for the baroreflex. The parasympathetic output comes from neurons in the nucleus ambiguus and from the dorsal motor nucleus. Parasympathetic preganglionic neurons possess elongated axons that synapse in close proximity to the target organ. Specifically, cardiac parasympathetic preganglionic neurons synapse onto postganglionic neurons situated within intrinsic cardiac ganglia [5,7]. The axons of these postganglionic neurons then project to extensive regions throughout the heart [8]. Transmission is mediated by the preganglionic release of Ach which binds to nicotinic acetylcholine receptors on the postganglionic neurons. Postganglionic neurons in turn release Ach, which binds to muscarinic receptors on cardiac myocytes and the coronary vasculature [7].

### 2.2. Sympathetic Innervation of the Heart

Sympathetic preganglionic neurons supplying the heart arise predominantly from the intermediolateral cell column of the upper thoracic spinal cord, mainly between T1 and T5 segments. These neurons are under tonic control from supraspinal autonomic centers, including the hypothalamus and brainstem nuclei such as the rostral ventrolateral medulla and medullary reticular formation (brainstem reticular formation), which integrate cardiovascular reflexes and central autonomic responses [7,9]. Preganglionic fibers leave the spinal cord through the ventral roots, enter the sympathetic chain, and synapse within cervical and upper thoracic paravertebral ganglia. Although the stellate ganglia have historically been considered the principal source of cardiac postganglionic sympathetic fibers, subsequent anatomical and electrophysiological studies have demonstrated important contributions from the superior cervical, middle cervical, thoracic, and mediastinal ganglia, as well as interactions with the intrinsic cardiac nervous system [1,5]. Postganglionic fibers then project to the SA node, atrioventricular node, atrial and ventricular myocardium, and coronary vasculature, providing region-specific modulation of cardiac performance [10]. Neurotransmission within sympathetic ganglia is mediated primarily by ACh acting on nicotinic receptors, whereas postganglionic terminals release NE, which binds mainly to β1-adrenergic receptors in cardiomyocytes and to α- and β-adrenergic receptors in vascular smooth muscle. Activation of these receptors initiates intracellular signaling pathways involving G proteins, cyclic adenosine monophosphate (cAMP), and protein kinase A, resulting in positive chronotropic, dromotropic, inotropic, and lusitropic effects [7,11,12]. Sustained sympathetic activation also promotes electrical instability, adverse remodeling, and arrhythmogenesis, highlighting its major role in cardiovascular disease states such as heart failure and ischemic heart disease [13,14]. In addition to efferent control, sympathetic pathways convey cardiac afferent signals from myocardial and coronary mechanoreceptors and nociceptors. These sensory fibers travel retrogradely through cardiac sympathetic nerves to dorsal root ganglia, predominantly at T1-T5 levels, before synapsing in the dorsal horn of the spinal cord. Ascending projections then relay information to medullary autonomic nuclei, thalamic centers, and cortical regions involved in cardiovascular reflex regulation and cardiac pain perception [5,15].

### 2.3. Sensory or Afferent Cardiac Innervation

Cardiac sensory neurons form a heterogeneous network of afferent pathways that continuously transmit information from the heart to both intrinsic and extrinsic components of the autonomic nervous system. This bidirectional signaling system plays a key role in cardiovascular reflex control and in higher-order autonomic regulation. Three main classes of cardiac afferent pathways are recognized: spinal afferents with cell bodies located in the dorsal root ganglia (DRG), vagal afferents with somata in the nodose ganglion, and intrinsic cardiac afferent neurons located within the intramural ganglionated plexuses of the heart, which are part of the intrinsic cardiac nervous system [1,13,14]. These neurons respond to mechanical, chemical, and ischemic stimuli, including changes in wall stress, intracardiac pressure, and metabolites released during myocardial ischemia. Spinal afferents, which predominantly travel alongside sympathetic nerves, have cell bodies in the upper thoracic DRG (T1–T5) and are mainly involved in nociceptive transmission, including ischemic cardiac pain [7]. Vagal afferents, in contrast, project through the nodose ganglion to central autonomic nuclei and primarily contribute to reflex regulation of cardiovascular homeostasis. Within the central nervous system, vagal afferents terminate in the nucleus tractus solitarius (NTS), which represents a key integrative hub for autonomic cardiovascular control. From the NTS, information is relayed to brainstem, hypothalamic, and cortical structures that modulate sympathetic and parasympathetic output [5,16]. Spinal afferents synapse in the dorsal horn of the spinal cord, from which ascending pathways convey nociceptive signals to higher brain centers involved in pain perception and autonomic responses [7]. The intrinsic cardiac ganglia further contribute to local integration of afferent and efferent signaling within the heart, supporting the concept of a distributed intrinsic cardiac nervous system capable of modulating regional cardiac function independently of central input [1]. This organization supports the concept of a bidirectional neurocardiac axis, in which sensory input from the heart continuously influences both reflex cardiovascular regulation and central autonomic network activity. Structurally, cardiac afferent fibers are not uniformly distributed within the myocardium. Experimental studies have shown that sympathetic-associated afferents course through epicardial regions before penetrating the myocardium, forming a functional epicardial-to-endocardial gradient [17]. Regional heterogeneity in innervation density has been described within the left ventricle, with relatively reduced innervation in basal inferior regions [17]. Disruption of this physiological organization may contribute to electrical instability and increased susceptibility to ventricular arrhythmias [17].

### 2.4. Cardiac Parasympathetic and Sympathetic Neurotransmission

#### 2.4.1. Cardiac Parasympathetic Neurotransmission and Cholinergic Signaling

**Figure 2:**
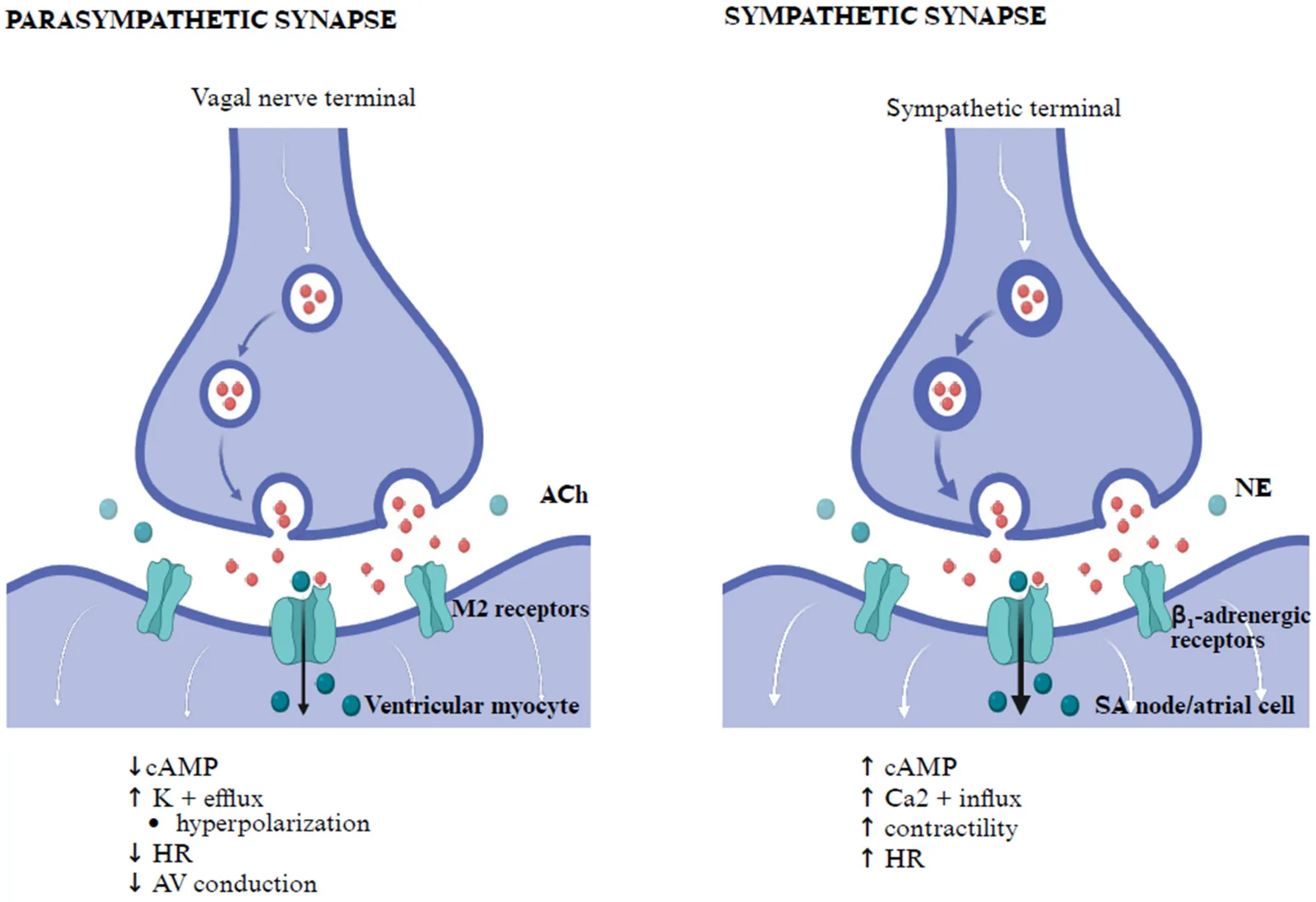
Cardiac parasympathetic and sympathetic neurotransmission. ACh: acetylcholine; AV: atrio-ventricular; cAMP: cyclic adenosine monophosphate; HR: heart rate; NE: norepinephrine.

Parasympathetic regulation of the heart is mediated primarily through the vagus nerve and postganglionic neurons located within the intrinsic cardiac ganglia. Preganglionic vagal fibers arise from medullary autonomic nuclei and synapse within ganglionated plexuses distributed in epicardial fat pads, particularly around the atria and great vessels [7,10]. Postganglionic neurons subsequently release ACh at the neuroeffector junction. ACh binds predominantly to muscarinic M2 receptors expressed in the SA node, atrioventricular node, and atrial myocardium. Activation of these receptors decreases heart rate, slows atrioventricular conduction, and reduces atrial contractility through inhibitory G proteins (Gi), which reduce adenylate cyclase activity, lower cAMP levels, and activate inwardly rectifying potassium channels (Figure 2). Parasympathetic effects on the ventricles are less pronounced because vagal innervation is denser in the atria and nodal tissue than in ventricular myocardium. Nevertheless, vagal activity exerts important antiarrhythmic and cardioprotective effects through modulation of sympathetic tone, HRV, and inflammatory pathways [13,18]. Termination of cholinergic signaling occurs through rapid hydrolysis of ACh by acetylcholinesterase.

#### 2.4.2. Cardiac Sympathetic Neurotransmission and Adrenergic Signaling

Sympathetic regulation of the heart is mediated by postganglionic nerve terminals that release norepinephrine (NE) at the neuroeffector junction. NE is synthesized from tyrosine, stored in presynaptic vesicles, and released by calcium-dependent exocytosis following neuronal stimulation [7,9]. Once released, NE binds to adrenergic receptors on cardiomyocytes, conduction tissue, and vascular smooth muscle, increasing HR, atrioventricular conduction, myocardial contractility, and relaxation (Figure 2). Presynaptic α2-receptors provide negative feedback by limiting further NE release [7]. Most released NE is rapidly cleared by neuronal reuptake through the norepinephrine transporter (NET; uptake-1 system), while a smaller fraction diffuses into the circulation and is metabolized by monoamine oxidase and catechol-O-methyltransferase [19]. Radiotracers such as ^123^I-metaiodobenzylguanidine (^123^I-mIBG) and ^11^C-metahydroxyephedrine (^11^C-mHED) can be used to assess cardiac sympathetic integrity and regional denervation [2,20]. Among cardiac adrenergic receptors, β1-receptors are predominant in the adult human heart, followed by β2-receptors, whereas β3-receptors are less abundant. β1-receptor stimulation is the principal mediator of positive chronotropic and inotropic responses through signaling pathways involving adenylate cyclase, cAMP, and protein kinase A [21]. Under chronic sympathetic activation, such as heart failure, persistent catecholamine exposure promotes receptor desensitization and downregulation through G-protein-coupled receptor kinases and β-arrestins, leading to reduced adrenergic reserve and impaired myocardial performance [22].

### 2.5. Molecular Control of Cardiac Autonomic Innervation

Cardiac autonomic innervation is regulated by coordinated signaling pathways that control both developmental patterning and post-injury neural remodeling. Dysregulation of these mechanisms contributes to heterogeneous sympathetic reinnervation and has been implicated in arrhythmogenic substrates following myocardial infarction. Neurotrophins, particularly nerve growth factor (NGF), are central to sympathetic neuron survival and axonal extension through TrkA-mediated signaling [23].

Cardiac NGF levels correlate with sympathetic nerve density, while experimental modulation of NGF results in proportional changes in cardiac innervation, ranging from hypoinnervation to hyperinnervation [24].

Spatial organization of cardiac nerves is additionally controlled by axon guidance molecules. Semaphorin-3A (Sema3A) acts as a major repulsive cue that shapes the transmural distribution of sympathetic fibers, contributing to the physiological epicardial-to-endocardial gradient. Altered Sema3A expression disrupts this pattern and modifies cardiac electrical stability [25]. Members of the glial cell line-derived neurotrophic factor (GDNF) family, including GDNF and artemin, further regulate autonomic development and post-injury remodeling. These factors promote sympathetic neurite outgrowth and functional neuro-cardiac connectivity, particularly under pathological conditions where they may exceed NGF-related trophic effects [26,27,28]. Overall, cardiac autonomic architecture results from the balance between neurotrophic (NGF, GDNF family) and repulsive (Sema3A) signaling pathways, whose disruption after injury contributes to maladaptive sympathetic remodeling and arrhythmogenesis [22,25,28].

Brain-derived neurotrophic factor (BDNF), another member of the neurotrophin family, has emerged as an additional regulator of neuronal plasticity and neuro-cardiovascular signaling. Unlike NGF, which primarily promotes sympathetic neuron survival and axonal growth through TrkA activation, BDNF predominantly influences synaptic maturation, neurotransmission, and activity-dependent neuronal remodeling through neurotrophin receptor signaling. Clinical studies have demonstrated altered BDNF signaling in cardiovascular disease, with reduced circulating BDNF levels observed in patients with heart failure and associated with disease severity and adverse clinical characteristics [29]. These findings were further supported by a recent systematic review and meta-analysis showing that circulating BDNF concentrations are consistently reduced in patients with heart failure compared with controls, suggesting impaired neurotrophic support in advanced cardiac dysfunction [30]. Experimental studies have demonstrated that BDNF can directly modulate sympathetic neurotransmission by inducing rapid changes in neurotransmitter release properties through p75 neurotrophin receptor (p75NTR)-dependent mechanisms, highlighting its role in activity-dependent autonomic plasticity [31].

## 3. Heart Transplantation and Cardiac Denervation

**Figure 3:**
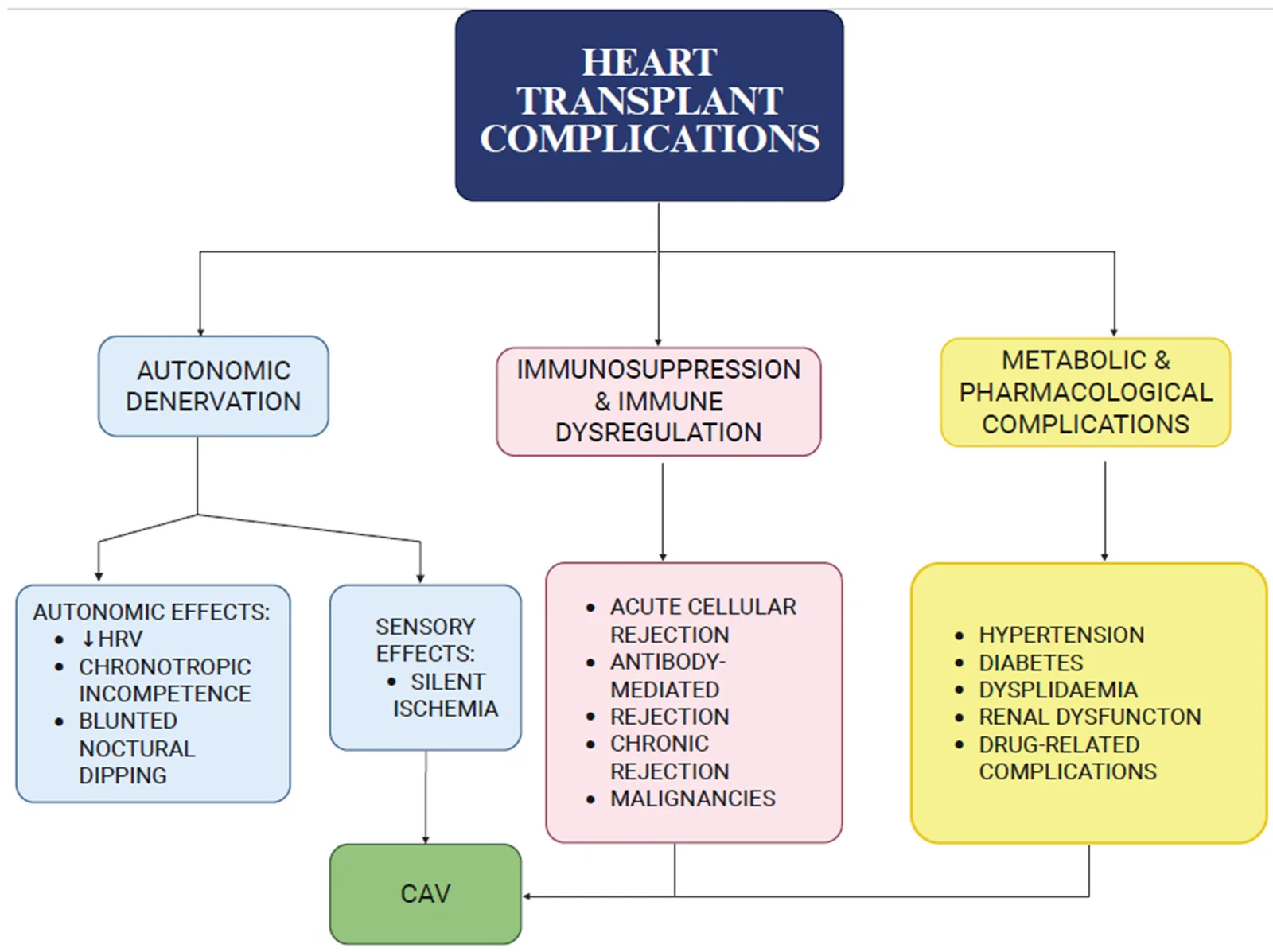
Summary flow chart of the main heart transplantation complications.

Heart transplantation represents the best therapeutic option for the treatment of patients with end-stage heart failure, as it provides better survival rates and quality of life scores [32]. Despite favorable surgical outcomes, HT remains associated with significant short- and long-term complications, that may negatively impact recipients’ outcomes [33]. These complications may be related to graft function and pathology or to diseases occurring in other organs mainly due to side effects of immunosuppressive drugs, and include acute and chronic rejection, CAV, opportunistic infections, hypertension, diabetes mellitus, renal dysfunction, malignancy and impaired autonomic regulation (Figure 3).

Cardiac transplantation is performed through a standardized sequence of donor organ procurement and recipient implantation. In the donor, the procedure is typically initiated via median sternotomy, followed by systemic heparinization and aortic cross-clamping. Antegrade infusion of cold crystalloid cardioplegic solution is then administered in the aortic root, combined with topical cooling to achieve rapid myocardial arrest and hypothermia. Cardiectomy is subsequently performed by dividing the superior and inferior venae cavae, pulmonary artery, aorta, and left atrial cuff, ensuring adequate vascular margins for implantation. Current organ-preservation strategies include static hypothermic storage as well as more advanced approaches such as hypothermic or normothermic machine perfusion, which aim to improve graft preservation and reduce ischemia–reperfusion injury [34]. Implantation of the graft in the recipient is performed via median sternotomy under cardiopulmonary bypass. The orthotopic heart transplantation technique was originally described by Lower and Shumway [35] and has since undergone several refinements. Among these, the bicaval technique [36] is currently the most widely used, as it is associated with improved haemodynamic performance, reduced incidence of tricuspid regurgitation, and better preservation of atrial geometry compared with the classical biatrial approach. This technique involves separate anastomoses of the superior and inferior vena cava, followed by reconstruction of the left atrial cuff, pulmonary artery, and ascending aorta.

Irrespective of the surgical technique employed, heart transplantation inevitably results in complete disruption of autonomic neural connections between the donor heart and the recipient’s nervous system. During procurement and implantation, all sympathetic and parasympathetic fibers innervating the myocardium are transected, leading to immediate and complete cardiac denervation in the early post-transplant period.

Under physiological conditions, cardiac function is tightly regulated by the autonomic nervous system through the integrated balance of sympathetic and parasympathetic inputs, which modulate HR, myocardial contractility, atrioventricular conduction, and cardiovascular adaptation to physiological and emotional stress. Following HT, the abrupt loss of neural regulation results in a characteristic pattern of functional alterations. These include an elevated resting HR, markedly reduced HRV, absence of rapid chronotropic response to exercise, and a predominant reliance on circulating catecholamines for cardiovascular adaptation to stress. Over time, partial and heterogeneous reinnervation may occur in some patients; however, early post-transplant physiology is defined by a state of functional cardiac denervation.

## 4. Heart Rate Variability

During HT, parasympathetic vagal pathways and intrinsic postganglionic sympathetic fibers projecting from the stellate ganglia to the myocardium are severed, resulting in Wallerian degeneration of the affected axons and consequent extrinsic cardiac denervation. The extent of autonomic disruption depends on the surgical technique employed. The biatrial technique interrupts only approximately half of the sympathetic neuronal connections while preserving most parasympathetic pathways, whereas the bicaval technique results in a more extensive and balanced interruption of both sympathetic and parasympathetic connections [37]. Because of this denervation, particularly during the early post-transplant period, cardiac function is primarily regulated by circulating hormones and intrinsic control mechanisms. Nevertheless, increasing evidence suggests that a degree of autonomic reinnervation may occur over time. This process can be assessed through several electrocardiographic parameters, including resting and exercise HR, HRV, and the low-frequency (LF) and high-frequency (HF) components of HRV. HT recipients typically exhibit higher resting HR than healthy individuals as a direct consequence of cardiac denervation. With time after transplantation, progressive autonomic reinnervation reduces the relative contribution of the intrinsic SA node rate, leading to a gradual decline in resting HR and a partial normalization of chronotropic control [38,39,40,41]. Another hallmark of HT recipients is an altered HR response to physical exercise. Compared with healthy controls and late HTx recipients, patients in the early post-transplant phase exhibit a slower and attenuated increase in HR during exercise [41,42,43]. Likewise, HR recovery after exercise is delayed in early HT recipients compared with both late recipients and healthy subjects [41,44].

Heart rate variability, defined as the beat-to-beat variation in HR, is widely used as a non-invasive marker of autonomic modulation because it reflects the balance between sympathetic and parasympathetic influences on the heart [45]. Due to the loss of autonomic innervation, HRV at rest and during exercise is significantly reduced in HT recipients compared with healthy individuals [46]. However, HRV progressively increases between approximately 3 months and 3 years after transplantation [47,48], suggesting the occurrence of autonomic reinnervation. This process appears to be heterogeneous and patchy, varying considerably among recipients [49]. Additional support for partial cardiac reinnervation comes from observations showing shorter RR intervals and reduced RR interval variability at rest in HT recipients [37]. The low-frequency component of HRV is considered an index of carotid baroreflex modulation of RR intervals and is commonly regarded as a marker of sympathetic and parasympathetic activity [46,50,51]. The LF component is markedly reduced in early HT recipients but approaches normal values in long-term recipients when compared with healthy controls [48,52,53,54]. These findings further support the occurrence of progressive reinnervation after HT.

The high-frequency (HF) (0.2 Hz) component of HRV is generally considered a marker of vagal activity and therefore reflects parasympathetic reinnervation after transplantation. Several studies have demonstrated significantly lower HF power in HT recipients than in healthy controls, indicating persistent impairment of parasympathetic function [46,55,56]. Nevertheless, evidence regarding parasympathetic reinnervation remains conflicting. Some investigations failed to detect signs of vagal reinnervation even 3–8 years after transplantation [57], whereas others reported evidence of parasympathetic recovery as early as 6 months to 2 years post-HT [47,57,58,59]. Although some studies suggest that HF power increases with time after transplantation, results remain inconsistent. Overall, available evidence indicates that parasympathetic reinnervation is a gradual and highly variable process that may be influenced by surgical factors, including the transplantation technique adopted [37]. Furthermore, parasympathetic reinnervation does not necessarily occur in parallel with sympathetic reinnervation in all heart recipients.

## 5. Cardiopulmonary Exercise Test

Cardiopulmonary Exercise Testing (CPET) enables an integrated assessment of the cardiovascular, pulmonary, and metabolic responses to exercise and therefore represents a valuable tool in the evaluation of HT recipients [60,61]. It provides unique insights into functional capacity [62,63], detection of allograft dysfunction [64,65], prognostication [66] and, intriguingly, into the non-invasive evaluation of cardiac reinnervation [67,68].

Compared with age- and sex-matched healthy individuals, exercise capacity in heart transplant recipients remains significantly reduced [61,62]. Although peak oxygen uptake (VO_2_ peak) improves after heart transplantation, patients still achieve only around 50–70% of the predicted value [69,70,71]. This limitation reflects several mechanisms, including both central (cardiac and pulmonary) and peripheral factors (endothelial dysfunction, skeletal muscle deconditioning and immunosuppressant-related myopathy) [72].

### 5.1. Chronotropic Response and the Denervated Heart

In the normal heart, exercise-induced vagal withdrawal and sympathetic activation induce a rapid and early increase in heart rate (HR). In the denervated transplanted heart, this neurally mediated response is absent; instead, HR rises more gradually and is primarily mediated by circulating catecholamines. Consequently, patients show an attenuated and delayed chronotropic response, an inability to achieve age-predicted maximal HR, and a typically flat HR-workload curve during incremental exercise [73]. The chronotropic response index (HR reserve relative to metabolic reserve) is consistently reduced, especially in the early post-transplant phase [74,75,76]. After cessation of exercise, HR may initially continue to rise, due to delayed catecholamine release, and subsequently show a blunted decline during the recovery phase as a result of absent vagal reactivation and circulating catecholamine metabolism.

### 5.2. Cardiac Reinnervation: Evidence from CPET

CPET can provide a range of indirect, yet simple and non-invasive, markers for the assessment of cardiac reinnervation in heart transplant recipients. Since the timing and extent of cardiac allograft reinnervation are heterogeneous, serial non-invasive assessments represent a valuable approach.

Heart transplant recipients with evidence of sympathetic reinnervation showed a significantly better exercise performance associated with an improvement in chronotropic and inotropic responses [77]. In these patients, exercise duration is longer, and both the rate of heart rate increase and peak heart rate during exercise are higher compared with patients without signs of reinnervation [77,78]. Indeed, a faster and proportionate heart rate response to incremental exercise can be considered the hallmark CPET signature of allograft sympathetic reinnervation, reflecting the restoration of adrenergic neural input to the SA node rather than sole reliance on circulating catecholamines [68,77]. In addition, studies have demonstrated that reinnervated recipients also exhibit greater HR reserve, a higher anaerobic threshold, faster HR recovery and possibly a higher peak VO_2_ compared to persistently denervated patients [67,68]. Oxygen pulse (VO_2_/HR), a non-invasive surrogate of stroke volume and contractile efficiency, may represent an additional CPET marker of cardiac re-innervation [79]. In reinnervated recipients, a more pronounced increase in oxygen pulse during exercise has been associated with greater exercise capacity and may be consistent with the enhanced inotropic response observed in patients with sympathetic reinnervation [77]. Moreover, it has been suggested that faster VO_2_ kinetics in the “cardiodynamic” phase (first 15–30 s of work) may indicate partial sympathetic re-innervation [80].

There are few data regarding functional impact of parasympathetic reinnervation on exercise physiology. Recovery parameters such as peak HR delay (delay in peak HR after exercise termination) and HR recovery at 2 min, which are influenced by parasympathetic activity, improved significantly within the first 2 years after heart transplantation [81].

The ability of CPET to non-invasively track the trajectory of functional status after HT has meaningful clinical implications [61,66,82]. Improvement in several parameters such as HR response and VO_2_ kinetics on serial CPET may provide non-invasive physiological evidence of cardiac re-innervation [67,68,78,79,80,81]. Recovery of allograft innervation is considered one of the key determinants of improved exercise capacity, which in turn is a significant predictor of post-transplant survival and quality of life [66,82,83].

## 6. Imaging of the Cardiac Autonomic System

The heart is supported by sympathetic activity in both physiologic and pathological conditions, through the release of NE, a neurotransmitter that binds to alpha- and beta-adrenoceptors and is subsequently cleared via presynaptic (uptake 1) and extra-neuronal transporters (uptake 2). After release, most of the NE in the synaptic space (50–80%) is removed by the presynaptic nerve terminal via the NE reuptake transporter (NET) in an energy-dependent manner (uptake 1). The NE then is repacked into secretory vesicles by the vesicular monoamine transporter (VMAT), while a small portion is metabolized by monoamine oxidase (MAO) and catechol-O-methyltransferase (COMT). The remaining NE is cleared by postsynaptic cells via an energy-independent mechanism (uptake 2), or diffuses into the vascular space [84]. Cardiac nuclear imaging allows for the non-invasive assessment of the functional integrity of cardiac sympathetic presynaptic terminals using NE-analogs radiopharmaceuticals.

^11^C-metahydroxyephedrine (^11^C-mHED), an isotope used for positron emission tomography (PET), is a NE analog that is rapidly transported into sympathetic neurons by the NET and stored in vesicles. PET imaging with ^11^CmHED has been extensively used to non-invasively characterize cardiac denervation and reinnervation process after HT. Initial studies showed that the reinnervation process is heterogenous, occurring initially in the left anterior descending artery (LAD) territory, later in the left circumflex territory, while the right coronary artery territory is substantially not involved in reinnervation [3,85]. The reinnervation proceeds in a basal to distal fashion from anterior to septal wall, then in the lateral wall, and lastly in the inferior wall and cardiac apex [3].

The likelihood of reinnervation is low within the first 12 to 18 months after HT but increases with time after HT, and once initiated, sympathetic reinnervation progressively increases even many years after HT [3,67]. However, the heart does not undergo full reinnervation even after 15 years after HT [3]. Retention of ^11^C-mHED has been observed in up to 59% of HT recipients [86]. The reinnervation depends on time after transplant and the higher HED retention has been found in HT recipients with idiopathic cardiomyopathy, younger age, younger donors’ age, fewer episodes of transplant rejection cases, shorter cross-clamp time and without diabetes [87,88].

A PET perfusion and sympathetic reinnervation study of HT recipients demonstrated that myocardial blood flow (MBF) is similar in all coronary territories despite differences in sympathetic reinnervation. However, in the territory with the highest uptake of ^11^C-mHED as the LAD territory, increase in MBF in response to sympathetic stimulation is higher compared to other territories with no evidence of reinnervation. These findings suggest that cardiac-efferent adrenergic signals play an important role in modulating MBF during activation of the sympathetic nervous system [89]. Other studies found that after HT denervated areas have increased basal glucose metabolism compared to reinnervated areas, despite having normal oxidative capacity and normal basal coronary flow [90], and reinnervated areas have appropriate increases in chronotropy and inotropy in response to aerobic exercise, while areas of denervation have blunted responses [77].

Preliminary data showed the feasibility of hybrid PET/magnetic resonance imaging (MRI) using ^11^C-mHED and demonstrated that cardiac reinnervation might have an influence on tissue composition [91]. No significant differences were found in wall motion (WM) and wall thickening (WT) between segments with or without reinnervation under resting conditions. However, myocardial segments with evidence of reinnervation by PET had non-significantly higher T_1_ relaxation times and a significantly higher mean extracellular volume (ECV), suggesting a higher percentage of diffuse fibrosis in these segments, without impairment of rest WM and WT [91].

^123^Iodine-meta-iodo-benzil-guanidine (^123^I-mIBG) scintigraphy is another nuclear imaging technique that allows the assessment of the functional integrity of postganglionic cardiac sympathetic innervation. The radiopharmaceutical ^123^I-mIBG is a NE analog that enters adrenergic nerve terminals via uptake 1 and is stored within synaptic vesicles. Because it escapes catabolism by both MAO and COMT, is serves as a robust diagnostic tool in nuclear medicine across oncology, neurology and cardiology [92]. In oncological applications, ^123^I-mIBG scintigraphy is primarily used to image neuroendocrine tumors, such as neuroblastoma and pheochromocytoma. In neurology, it aids in differentiating Parkinson’s disease from other conditions like dementia and Lewy body dementia, by identifying postganglionic sympathetic dysfunction. In cardiology, ^123^I-mIBG scintigraphy is used to assess cardiac sympathetic innervation, particularly in patients with heart failure, ischemic heart disease, diabetic cardiomyopathy, cardiac amyloidosis and arrhythmias, providing prognostic information that contributes to risk stratification of the patient and may guide treatment strategies [93]. Cardiac ^123^I-mIBG image findings strongly correlate with the severity and prognosis of many cardiovascular diseases, especially heart failure, where the ability to predict occurrence of potentially fatal arrhythmias may help with the better selection of patients who will benefit from an implantable cardioverter defibrillator [94]. Similarly, in patients with ischemic heart disease, cardiac ^123^I-mIBG imaging provides information on the extent of damage and identifies arrhythmogenic regions.

Following intravenous administration, ^123^I-mIBG is actively transported into cardiac sympathetic nerves via the NET and stored within synaptic vesicles. Consequently, the myocardial distribution and retention of ^123^I-mIBG directly reflect the functional integrity of the sympathetic nervous system. Cardiac ^123^I-mIBG scintigraphy involves planar acquisitions used to evaluate global cardiac ^123^I-mIBG uptake and single-photon emission tomography (SPECT) imaging to assess the regional distribution of the radiotracer. Scintigraphic images are acquired at distinct intervals after injection. Early images, typically captured 15–30 min after administration, reflect the initial tracer distribution. In contrast, delayed images are obtained 3–4 h post-injection to evaluate the retention and washout kinetics of ^123^I-mIBG. Ultimately, the heart-to-mediastinum ratio (H/M ratio) and the washout rate serve as the primary quantitative parameters to assess cardiac sympathetic innervation. The early and late H/M ratio, derived 15 min and 4 h post-injection, are both considered to be markers of NE uptake; the washout rate is calculated according to the following formula: WO = [(Early H/M ratio- late H/M ratio)/(early H/M ratio)] × 100 and is a marker of NE turnover/sympathetic tone. A reduced H/M ratio paired with an elevated washout rate indicates impaired sympathetic innervation. Diminished cardiac uptake of ^123^I-mIBG has been correlated with a poor prognosis in heart failure patients, predicting an increased risk of all-cause mortality and adverse cardiac events [95,96].

Cardiac ^123^I-mIBG scintigraphy studies confirmed that reinnervation after HT transplant occurs after the first year post-transplantation and progressively increases over time, beginning from the base of the heart and spreading toward the apex. Uptake is primarily observed in the anterior, anterolateral and septal regions, while ^123^I-mIBG is not apparent in the posterior or inferior myocardial regions, except for some basal posterior localization. A study conducted on 31 HT patients without diabetes mellitus, neuropathies or active rejection, from 6 months to 12 years post-transplantation, showed myocardial ^123^I-mIBG uptake in 70% of the recipients [97]. The H/M ratio correlated positively with time after transplantation: patients studied from 2 to 12 years post-transplantation had an H/M ratio significantly higher than patients studied before 2 years post-transplantation. Although ^123^I-mIBG uptake progressively increases with time after transplantation, global innervation was not observed even up to 12 years post-transplantation. Myocardial ^123^I-mIBG uptake was anterolateral in 16 patients, anterior in three and anterolateral and septal in three. Eight patients developed CAV, and five of them had decreased myocardial ^123^I-mIBG uptake, suggesting that vasculopathy may delay the process of sympathetic reinnervation. Relative ischemia resulting from vascular rejection may be associated with lower likelihood of nerve regeneration, similarly to non-transplanted hearts, in which ischemic episodes are known to result in nerve damage and low ^123^I-mIBG uptake [97]. Another study including 12 HT recipients and 14 healthy controls show that 50% of the recipients had H/M ratio > 1.3 indicating cardiac sympathetic reinnervation on ^123^I-mIBG scintigraphy [53]. Simultaneous ventricular sympathetic reinnervation assessed by ^123^I-mIBG scintigraphy and SA node sympathetic reinnervation assessed by HRV were detected only in two patients (17%), suggesting that eventual SA node sympathetic reinnervation and left ventricular sympathetic reinnervation do not occur simultaneously. Additionally, ventricular reinnervation process was time-dependent while SA node reinnervation was not [53]. Interestingly HT recipients with significant ^123^I-mIBG uptake had improved exercise tolerance, longer duration of exercise, and higher maximum VO_2_ compared to recipients with lower ^123^I-mIBG uptake [98]. Additionally, HT recipients with higher H/M ratio had a higher relative density of myocardial nerve fibers by use of the PGP 9.5 neuronal marker [98].

Recent reports showed that ^18^F-meta-fluorobenzylguanidine (^18^F-mFBG) is an emerging PET radiotracer for the assessment of myocardial sympathetic innervation [99]. Like ^123^I-mIBG, ^18^F-mFBG is a benzylguanidine analog, whose uptake in the presynaptic nerve terminus is mediated by the NET. Compared to ^123^I-mIBG scintigraphy, ^18^F-mFBG PET imaging offers a higher spatial resolution and visual quality, an absolute quantification of nerve density and reinnervation instead of semi-quantitative results, providing the exact percentage of functioning nerve terminals per gram of heart tissue and the possibility to track nerve fiber regeneration over time, faster procedures (1 h vs. 3–4 h) and lower radiation doses. To date, no studies have been published yet on cardiac reinnervation after HT using ^18^F-mFBG PET; however, this imaging technique appears to be promising for the evaluation of cardiac sympathetic nerve integrity and function.

## 7. Immunohistochemical Markers of Cardiac Reinnervation

Orthotopic heart transplantation entails complete transection of the donor heart’s nerves and therefore autonomic denervation of the implanted organ. Numerous functional studies using neurochemical, nuclear imaging and electrophysiological methods have documented that reinnervation, predominantly sympathetic, can develop progressively in the years following surgery, and that it is associated with improved oxygen uptake, HR and contractile function during exercise [77]. This reinnervation is, however, heterogeneous both regionally and temporally, with wide interindividual variability [100].

Direct morphological confirmation of the presence and distribution of nervous tissue in the myocardium requires immunohistochemistry on endomyocardial biopsies or autopsy specimens [101,102].

Immunohistochemistry allows nerve fibers to be identified within myocardial tissue by means of antibodies directed against specific neural proteins, applied to formalin-fixed, paraffin-embedded tissue sections after antigen retrieval (citrate buffer, microwave heating), blocking of endogenous peroxidases and incubation with the primary antibody; nerve density is then quantified as the number of nerves per mm^2^ in the different areas of interest, such as endocardium, mid-myocardium and perivascular sites [102].

The markers used reflect distinct cellular and functional components of nervous tissue. S-100 protein, expressed by Schwann cells, is used as a pan-neural marker to reveal the overall structure of nerves [102,103]. Tyrosine hydroxylase (TH), the rate-limiting enzyme of catecholamine synthesis, selectively labels sympathetic (adrenergic) fibers and represents the principal indicator of functionally relevant sympathetic reinnervation [102,103,104]. Growth-associated protein GAP-43, induced in axons undergoing extension, identifies growth cones and is therefore a marker of nerve sprouting and active axonal regeneration [103]. Additional pan-neuronal markers include the neuron-specific protein gene product 9.5 (PGP9.5) and 200 kD neurofilaments, which allow assessment of neuronal integrity and of the basic structural architecture of the fibers, respectively [103]. The combined use of S-100 and TH makes it possible to distinguish the overall nerve population from the adrenergic component alone, since S-100 also labels supporting glial elements and non-adrenergic fibers, whereas TH is specific for catecholaminergic neurons [102]. Parasympathetic (cholinergic) fibers can be assessed with markers such as acetylcholinesterase or choline acetyltransferase, although studies of the transplanted heart have focused mainly on the sympathetic component. The combination of these antibodies provides complementary information on the dynamics of denervation and reinnervation. In serial endomyocardial biopsies performed in the first months after transplantation, in normal myocardium, the immunoreactive fibers run in the interstitium around vessels and in close contact with the myofibrils; after transplantation the immunolabeling for PGP9.5, S-100 and TH disappears during the first month, while the basic nerve structure revealed by neurofilaments remains preserved. Immunoreactivity reappears during the second month, initially in the interstitial space around blood vessels, with TH and PGP9.5 detectable between the fourth and sixth week and S-100 only later and in a minority of patients, independently of rejection episodes [103]. In longer-term autopsy series, the total number of S-100-positive nerves tends to decrease over time, whereas GAP-43-positive fibers in the perivascular region increase progressively, reflecting active and persistent nerve sprouting; TH-positive fibers are abundant, confirming the sympathetic nature of the reinnervation, which is more marked in patients transplanted for ischemic heart disease than in those with dilated cardiomyopathy [102]. Immunohistochemical and immunofluorescence analysis also allows study of the spatial relationships between nerve fibers and the other structures of the myocardium. Recent studies, combining TH with endothelial markers such as CD34, have shown that a substantial fraction of sympathetic terminals lies in close apposition to the capillary network, defining a cardiac neurovascular unit. In the transplanted heart, denervation is associated with early and persistent capillary remodeling, with reduced vessel caliber [104].

Overall, immunohistochemistry is the method of choice to provide direct histological proof of the chronological sequence of denervation and subsequent reinnervation, to characterize its component (sympathetic, sensory or glial supporting) and topographic distribution, and to correlate these findings with functional data and with the structural and microvascular remodeling of the transplanted heart.

## 8. Clinical Implications of Cardiac Denervation and Reinnervation

Donor heart procurement, recipient cardiectomy and graft surgical implantation imply sudden and unavoidable cardiac denervation which has multiple consequences on HT recipients, including abnormally elevated HR, reduced HRV, compromised exercise capacity, altered blood pressure control and lack of chest pain [105]. Autonomic denervation secondary to the transection of nerve fibers during HT fundamentally alters cardiac chronotropic regulation. The loss of parasympathetic vagal tone abolishes the physiological suppression of the SA node, resulting in a characteristically resting HR. Simultaneously, sympathetic denervation induces a delay in exercise or stress-induced augmentation of SA node automaticity, resulting in reduced maximal HR response and increase in cardiac output with exercise, with adrenergic stimulation of the heart depending entirely on circulating catecholamines.

Heart recipients experience a delayed achievement of resting HR during the recovery phase after exercise, because the HR continues to rise after cessation of exercise due to a delayed humoral catecholamine release. Additionally, catecholamine storage depletion in the denervated myocardium and reliance on circulating catecholamine also result in increased β-adrenergic receptor expression and hypersensitivity to catecholamines that may increase the risk of ventricular arrhythmia and sudden cardiac death [106]. The transplanted heart may increase its cardiac output during exercise by increasing stroke volume through the Frank–Starling mechanism. Although intrinsic cardiac impulse formation and conduction are intact, the donor-denervated heart is critically pre-load-dependent and higher left ventricular filling pressures are needed, particularly during exercise. However, only rarely can HT recipients achieve a normal exercise capacity as assessed by peak VO_2_ levels. Cardio-pulmonary and arterial baroreflex responses are also impaired after HT, affecting diurnal blood pressure variability and blunting nocturnal fall in blood pressure, therefore contributing to hypertension and cardiac hypertrophy. A study conducted on 50 HT recipients demonstrated higher blood pressures and total peripheral resistance at supine rest compared to healthy subjects. These patients also exhibited attenuated blood pressure and total peripheral resistance responses during orthostatic challenge and preserved blood pressure and total peripheral resistance responses, but a reduced cardiac output response during isometric exercise [107].

Previous studies showed that basal coronary blood flow at rest is increased compared to healthy subjects during the first year post-HT, and then it gradually declines to reach levels similar to those observed in healthy individuals after 3 years of HT. The absence of α-adrenergic constriction of the coronary circulation in the denervated hearts could explain why basal myocardial blood flow is elevated in the first year following HT and then declined over time with sympathetic reinnervation [108]. Myocardial flow reserve and maximal dilator capacity, assessed with adenosine injection, remain stable during the 3 years post-HT, and then decline due to an increase in coronary vascular resistance, that may be attributed to immune-mediated endothelial injury and dysfunction and with the development of CAV [108]. Additionally, basal coronary blood flow has been demonstrated to be similar in all coronary regions; however, after sympathetic nerve stimulation, blood flow has been shown to increase in different manners at different coronary territories, with a greater increase in flow in the LAD territory where maximal sympathetic reinnervation occurs [3]. These finding suggest that restoration of sympathetic reinnervation can enhance the regulation of myocardial blood flow, and that the increase in coronary flow in response to sympathetic stimulation correlates with the density of regional NE stores within cardiac sympathetic nerve terminals.

Sympathetic nerves innervate coronary resistance vessels and can affect tone through mechanisms directly on vascular smooth muscle cells as well as by stimulating the release of nitric oxide (NO) from the endothelium. The effects of sympathetic activation on myocardial perfusion and coronary resistance vessel tone are complex and dependent on the net actions of beta-1 (β_1_)-mediated increases in myocardial contractility and O_2_ consumption, direct β_1_ (NO linked) and β_2_-mediated coronary resistance vessel dilation, and α_1_-mediated constriction [109]. Under normal healthy conditions, coronary dilatation predominates due to exercise-induced β-adrenergic receptor stimulation, resulting in an increase in flow that is commensurate with the increase in myocardial O_2_ consumption.

The absence of afferent and efferent heart innervation after HT also eliminates nociceptive signaling, precluding typical angina in heart recipients. The inability to perceive pain does not allow symptomatic recognition of CAV and HT recipients often develop acute ischemic events or left ventricular dysfunction or die suddenly. Rejection often manifests as isolated diastolic dysfunction or silent ischemia, necessitating routine surveillance via serial endomyocardial biopsies and coronary angiography to detect microvascular injury and subclinical coronary disease.

Over time, both sympathetic and parasympathetic reinnervation will occur, although the extent of this process remains incomplete, non-uniform, variable across patients cohorts, and heterogeneous within the same patient. Cardiac reinnervation of the transplanted heart may develop in 40% to 70% of heart recipients [37,53,97,98], with patients with CAV [98], diabetes [87] and acute rejection [88] showing a lesser degree of reinnervation. Although most studies confirm complete denervation within the first 6–12 months post-transplantation, subsequent sympathetic re-innervation is progressive and continues to increase even during the late post-operative period, extending up to 15 years after surgery, with time following HT significantly correlating with the extent of reinnervation [87].

It is worthy of note that full reinnervation of the heart has not been shown even after 15 years of HT [97] and some patients demonstrated no evidence of reinnervation even 10–13 years after transplantation [67]. Sympathetic reinnervation occurs earlier than parasympathetic reinnervation, with previous studies showing that signs of sympathetic reinnervation emerged within 6 months, while signs of parasympathetic reinnervation emerged by 24 months [110].

Studies using microscopy revealed that sympathetic nerves grow along both the coronary vasculature and the surgical anastomoses between the donor graft and the recipient remaining heart tissue [111].

Although extremely heterogenous, immunohistochemical [101] and non-invasive imaging studies [77] have revealed that reinnervation proceeds in a base-to-apex direction, from epicardium to endocardium, mirroring the distribution of the coronary vasculature, and is more evident in the left ventricular anterior, and septal wall is reinnervated earlier, whereas lateral wall is reinnervated later. Lack of reinnervation in the inferior myocardial regions may partially explain the inability of morphologic studies to identify increases in neuronal density in right ventricular endomyocardial biopsy specimens.

Heart recipients with evidence of restored sympathetic innervation exhibit enhanced exercise performance compared to their denervated counterparts, a benefit mediated by superior chronotropic and inotropic responses [77]. The reinnervation process significantly correlates with an increase in ejection fraction (EF) during exertion, driven primarily by improvements in the anteroseptal regional EF. Notably, this regional enhancement aligns with the vascular territory of the LAD artery where the reinnervation process typically initiates. Conversely, the infero-apical region remains denervated in the majority of HT recipients, resulting in a concurrently lower regional peak EF [77]. The improved exercise capacity observed in reinnervated heart recipients also provides them better quality of life [88].

A long-term longitudinal study involving 14 heart recipients followed for 10 years after transplant showed a significant increase in HRV during the follow-up period associated with a parallel increase in LF and HF components [111]. The HF component of the HRV is usually considered as the expression of vagal activity and is correlated to respiratory sinus arrythmia, while the LF component is dependent on both sympathetic and parasympathetic activity and baroreflex responses. Therefore, these results suggest a partial re-innervation of the sinus node [112].

Cardiac allograft vasculopathy represents a leading cause of mortality after HT and affects 30% and 50% of HT recipients at 5 and 10 years respectively. It is caused by allograft endothelial injury due to immune and inflammatory factors causing intimal hyperplasia and luminal narrowing [113]. Early diagnosis of CAV is critically limited by the absence of ischemic symptoms in the denervated allograft, necessitating annual surveillance angiography to detect early coronary involvement. To date, few studies have investigated the relationship between the extent of cardiac reinnervation and the development of CAV. It is hypothesized that, in many heart recipients, CAV progression occurs simultaneously with the reinnervation process. Consequently, NO release may be affected, not only by adrenergic influences but also by impairment of endothelial cell function [82]. Equally significant may be the reciprocal effect of CAV on the reinnervation process itself. Previous investigations evaluating the extent of reinnervation and vasculopathy in 31 HT recipients revealed that patients with established angiographic CAV exhibited significantly less reinnervation as assessed by ^12^^3^I-mIBG imaging compared to those without CAV [97].

Although there is accumulating evidence that areas of reinnervated myocardium have improved blood flow regulation, energy substrate use, cardiac performance, and exercise capacity, associations between reinnervation and improved survival remain scarce. A recent published study reported on 232 HT recipients tested once for the presence of sympathetic reinnervation of the sinus node using the LF component of HRV, and then followed over a period of 27 years after transplant [114]. The prevalence of sympathetic reinnervation was 70% and it primarily occurred in the first 18 months after transplant. Sympathetic reinnervation was associated with a significant survival benefit, yielding a median survival time of 19.9 years for reinnervated patients compared to 14.4 years for the denervated cohort [114].

Spatial and temporal heterogeneity in sympathetic reinnervation may lead to asymmetric or incomplete neural recovery across the heart and create a pro-arrhythmic substrate that may enhance the risk of severe ventricular arrhythmias and sudden cardiac death, as highlighted by studies conducted in patients with heart failure and ischemic heart disease [94]. Therefore, assessing the symmetry and completeness of sympathetic reinnervation with non-invasive techniques as HRV analysis, CPET and PET imaging is crucial for risk stratification and prevention of adverse events in these patients.

## 9. Future Perspectives

The improved exercise capacity, long-term survival and quality of life associated with cardiac reinnervation after HT highlight the potential of stimulating reinnervation or re-establishing nervous connections to further optimize heart recipients’ outcomes (Table 2).

Nerve growth factor is known to enhance reinnervation of surgically denervated canine heart [115] and experimental studies showed that NGF administration to the stellate ganglia can cause significant cardiac nerve sprouting [116]. In dogs, myocardial infarction (MI) results in immediate local NGF release, followed by upregulation of NGF and GAP43 expression from nonneuronal cells at the infarcted site [117]. NGF and GAP43 are transported retrogradely to the stellate ganglion, triggering nerve sprouting at the non-infarcted left ventricular free wall and, to a lesser degree, the infarcted site. Individual differences in NGF expression might be partially responsible for differential nerve sprouting and susceptibility to arrhythmia after MI [117]. It has been hypothesized that NGF could ameliorate cardiac reinnervation, through endothelial cells engineered to produce NGF engrafted in the transplanted heart [118]. However, concerns still remain about the possibility that NGF could entail a sympathetic hyperinnervation with limited effect on parasympathetic neurons growth that could trigger fatal arrhythmias [119].

Experimental studies have demonstrated that early vagal reconnection following transection at the cardiac level utilizing an implantable chitosan/poly-ε-caprolactone cuff-like nerve guidance conduit preserves myocardial mechanical function and enhances global circumferential, longitudinal, and radial strains and attenuated diastolic desynchrony [120]. Although histological analysis revealed only partial structural repair with approximately 20% viable vagal fascicles, the functional restoration of myocardial parasympathetic fibers is sufficient to normalize oxidative stress and prevent interstitial fibrosis. These findings suggest that preserving even limited vagal input mitigates early adverse cardiac remodeling. Surgical reconnection of the right vagus nerve via a chitosan/poly-ε-caprolactone cuff-like nerve guidance conduit may represent a novel therapeutic strategy to prevent right vagotomy-induced heart failure following major thoracic surgery or cardiothoracic transplantation [120].

Implantable neural interfaces targeting the central and peripheral nervous systems are currently utilized to restore sensory, motor, and cognitive functions in individuals with disabilities, yielding highly promising outcomes. Furthermore, these interfaces have been increasingly deployed to modulate autonomic activities as a therapeutic intervention for chronic metabolic and cardiovascular diseases as diabetes and hypertension. The application of these technologies, also called “organ neuroprostheses”, could be extended to re-establish the connection between transplanted organs and the host nervous system, thereby enhancing the long-term efficacy and the functional biointegration of the grafts [121]. Specifically, restoring the regional vagal connection through the development of a closed-loop organic neuroprosthetic system holds the potential to significantly improve the overall long-term success of HT. Utilization of a closed-loop system based on continuous simultaneous recording of ventricular stiffness and donor heart vagal activity reconnected to the recipient’s vagus nerve could precisely trigger vagal stimulation. This stimulation could prevent disruptions to the delicate balance between sympathetic and parasympathetic influences, ultimately mitigating the risk of critical cardiac complications, such as heart failure, arrhythmias, and endothelial dysfunction [121].

## 10. Conclusions

Heart transplantation results in surgical denervation of the donor heart, with complete loss of sympathetic and parasympathetic systems. Relationships between reinnervation and CAV, risk of arrhythmias, exercise capacity and survival should be interpreted with caution, since the evidence comes mostly from small, observational and heterogeneous cohorts. Potential confounding factors may influence the assessment of reinnervation as recipient age, duration of transplantation, presence/absence of diabetes, rejection, technique of surgery, medications, donor characteristics, physical training and comorbidities. Prospective and long-term follow-up studies on a larger number of patients are warranted to evaluate the clinical consequences of denervation and reinnervation of the graft, as well as to identify modifiable factors that can affect the reinnervation process. Increased knowledge of the reinnervation process could improve understanding of pathophysiologic mechanisms underlying post-transplant complications, such as CAV, endothelial dysfunction, hypertension and arrhythmias. Advancements in non-invasive techniques for the assessment of cardiac denervation/reinnervation after transplantation are required to better define the risk-stratification of such patients. Finally, further investigation is needed to actively induce optimal reinnervation in HT patients, thereby improving exercise tolerance, long-term survival, and most importantly, quality of life.

## Figures and Tables

**Table 1:** Comparison between the different techniques used for the assessment of cardiac reinnervation after heart transplantation.

Technique	Physiological Target	Main Parameter(s)	Spatial Resolution	Advantages	Disadvantages	Invasiveness	Level of Evidence
HRV	Global autonomic balance (LF ≈ sympathetic, HF ≈ parasympathetic)	LF power HF power LF/HF ratio resting HR	None (whole-heart global signal)	Non-invasive Inexpensive Bedside Repeatable/serial follow-up	Indirect (functional, not anatomical) Parasympathetic findings inconsistent across studies	None	Moderate
CPET	Functional sympathetic reinnervation Indirect parasympathetic (HR recovery)	HR-workload slope chronotropic index VO_2_ peak O_2_ pulse HR recovery	None (global functional response)	Non-invasive Integrated cardiopulmonary and prognostic data	Indirect/no anatomical localization Confounded by peripheral/muscular factors	None	Moderate
^11^C-mHED PET	Regional sympathetic nerve terminal integrity	Regional tracer uptake/retention	High—territorial/regional mapping (LAD vs. circumflex vs. RCA territory)	High spatial resolution Regional mapping Combinable with perfusion/MRI	High cost Limited availability Cyclotron-dependent Short half-life	Low	Moderate
^123^I-mIBG scintigraphy	Postganglionic sympathetic innervation	H/M ratio washout rate	Moderate—regional (SPECT) Lower resolution than PET	Wider availability than PET Established prognostic value	Semi-quantitative Lower spatial resolution Long protocol (3–4 h)	Low	High
Immunhistochemistry	Direct histological nerve presence/density (sympathetic, pan-neural, regenerating)	Nerve density per mm^2^ (S-100, TH, GAP-43, PGP9.5)	Site-specific (limited to EMB sampling site, not whole-heart)	Direct histological proof Distinguishes fiber subtypes	Invasive Sampling error (focal/patchy reinnervation)	High (EMB)	Low

CPET: cardiopulmonary exercise testing; EMB: endomyocardial biopsy; GAP-43: growth-associated protein 43; HED: hydroxyephedrine; HF: high frequency; H/M: heart-to-mediastinum; HR: heart variability; HRV: heart rate variability; LAD: left anterior descending; LF: low frequency; mIBG: metaiodobenzylguanidine; MRI: magnetic resonance imaging; PET: positron emission tomography; PGP9.5: protein gene product 9.5; RCA: right coronary artery; SPECT: single-photon emission computed tomography; TH: tyrosine hydroxylase; VO_2_: oxygen uptake.

**Table 2:** Summary of the major clinical trials/cohorts linking cardiac reinnervation after heart transplantation to recipients’ exercise capacity, cardiac allograft vasculopathy and long-term survival.

Study, Year, Ref.	Population	Diagnostic Method	Outcome Domain	Key Finding
Parry et al., 1997 [98]	34 HT recipients 12–132 months after HT	^123^I-mIBG scintigraphy CPET Immunohistochemistry	Exercise capacity	Higher ^123^I-mIBG uptake associated with improved exercise tolerance, longer exercise duration, higher peak VO_2_, and higher myocardial nerve fiber density.
Di Carli et al., 1997, [89]	14 HT recipients vs. 8 healthy controls	^11^C-mHED PET	Coronary/graft hemodynamics	Resting MBF similar across territories regardless of reinnervation status MBF increase in response to sympathetic stimulation was greater in the reinnervated territory.
Bernardi et al., 1998, [37]	89 HT recipients 79 Biatrial technique vs. 10 Bicaval technique	HRV	Surgical technique/parasympathetic reinnervation	Biatrial technique interrupts 50% of sympathetic connections while preserving most parasympathetic pathways Bicaval technique causes more extensive, balanced interruption of both sympathetic and parasympathetic connections influencing the pattern of later parasympathetic reinnervation.
Estorch et al., 1999 [97]	31 HT recipients 6 month–12 years after HT Exclusion criteria: diabetes, neuropathy, active rejection	^123^I-mIBG scintigraphy	CAV	70% HT recipients showed myocardial uptake H/M ratio increased with time post-transplant 8 patients developed CAV, 5 had decreased uptake—suggesting CAV may delay/impair sympathetic reinnervation.
Bengel et al., 2001 [77]	29 HT recipients vs. 10 healthy controls	^11^C-mHED PET CPET	Exercise performance/graft hemodynamics	Reinnervated regions showed appropriate chronotropic/inotropic increases during exercise Reinnervation correlated with increased ejection fraction during exercise, mainly in the anteroseptal region.
Lovric et al., 2004 [53]	12 HT recipients vs. 14 healthy controls	^123^I-mIBG scintigraphy HRV	Reinnervation concordance (SA node vs. ventricle)	50% HT recipients had H/M ratio >1.3 Concordant SA-node and ventricular reinnervation in only 17% of patients Ventricular reinnervation was time-dependent, SA-node reinnervation was not.
Beitzke et al., 2019 [91]	10 HT recipients 4–21 years after HT	Hybrid PET/MRI	Structural/tissue correlate	Reinnervated segments had higher extracellular volume and T1 relaxation time (indicating more diffuse fibrosis), without impairment of resting wall motion or wall thickening.
Weiner et al., 2025 [114]	232 HT recipients tested once for sinus-node sympathetic reinnervation, followed for up to 27 years	HRV (LF component)	Long-term survival	Prevalence of sympathetic reinnervation was 70%, occurring primarily within the first 18 months post-HT Reinnervation was associated with significantly improved survival: median survival 19.9 years vs. 14.4 years in patients without reinnervation.

CAV: cardiac allograft vasculopathy; CPET: cardiopulmonary exercise testing; HED: hydroxyephedrine; HF: high frequency; H/M: heart-to-mediastinum; HRV: heart rate variability; HT: heart transplantation; MBF: myocardial blood flow; mIBG: metaiodobenzylguanidine; MRI: magnetic resonance imaging; PET: positron emission tomography; SA: sinoatrial; VO_2_: oxygen uptake.

## Data Availability

No new data were created or analyzed in this study.

## Abbreviations

The following abbreviations are used in this manuscript:

Ach	Acetylcholine
BDNF	Brain derived neutrophic factor
BSFR	Brainstem Reticular Formation
cAMP	Cyclic Adenosine Monophosphate
CAV	Cardiac Allograft Vasculopathy
CPET	Cardio-pulmonary Exercise testing
DRG	Dorsal Root Ganglia
EF	Ejection fraction
EMB	Endomyocardial Biopsy
FBG	Fluoro-benzyl-guanidine
GAP43	Growth-associated protein 43
GDNF	Glial cell line-derived neurotrophic factor
HED	Hydroxyephedrine
HF	High frequency
H/M	Heart-to-mediastinum
HRV	Heart Rate Variability
HT	Heart Transplantation
LAD	Left anterior descending
LF	Low frequency
MBF	Myocardial blood flow
MIBG	Metaiodobenzylguanidine
MRI	Magnetic resonance imaging
NE	Norepinephrine
NGF	Nerve growth factor
NO	Nitric oxide
NTS	Nucleus tractus solitarius
PET	Positron emission tomography
PGP 9.5	Protein gene product 9.5
SA	Sinoatrial
SPECT	Single-photon emission computed tomography
TH	Tyrosine hydroxylase
VO_2_	Oxygen uptake
